# Identification of Maize Kernel Vigor under Different Accelerated Aging Times Using Hyperspectral Imaging

**DOI:** 10.3390/molecules23123078

**Published:** 2018-11-25

**Authors:** Lei Feng, Susu Zhu, Chu Zhang, Yidan Bao, Xuping Feng, Yong He

**Affiliations:** 1 College of Biosystems Engineering and Food Science, Zhejiang University, Hangzhou 310058, China; lfeng@zju.edu.cn (L.F.); sszhu@zju.edu.cn (S.Z.); chuzh@zju.edu.cn (C.Z.); ydbao@zju.edu.cn (Y.B.); pimmmx@163.com (X.F.); 2 Key Laboratory of Spectroscopy Sensing, Ministry of Agriculture and Rural Affairs, Hangzhou 310058, China; 3 State Key Laboratory of Modern Optical Instrumentation, Zhejiang University, Hangzhou 310058, China

**Keywords:** maize kernel, hyperspectral imaging technology, accelerated aging, principal component analysis, support vector machine model, standard germination tests

## Abstract

Seed aging during storage is irreversible, and a rapid, accurate detection method for seed vigor detection during seed aging is of great importance for seed companies and farmers. In this study, an artificial accelerated aging treatment was used to simulate the maize kernel aging process, and hyperspectral imaging at the spectral range of 874–1734 nm was applied as a rapid and accurate technique to identify seed vigor under different accelerated aging time regimes. Hyperspectral images of two varieties of maize processed with eight different aging duration times (0, 12, 24, 36, 48, 72, 96 and 120 h) were acquired. Principal component analysis (PCA) was used to conduct a qualitative analysis on maize kernels under different accelerated aging time conditions. Second-order derivatization was applied to select characteristic wavelengths. Classification models (support vector machine−SVM) based on full spectra and optimal wavelengths were built. The results showed that misclassification in unprocessed maize kernels was rare, while some misclassification occurred in maize kernels after the short aging times of 12 and 24 h. On the whole, classification accuracies of maize kernels after relatively short aging times (0, 12 and 24 h) were higher, ranging from 61% to 100%. Maize kernels with longer aging time (36, 48, 72, 96, 120 h) had lower classification accuracies. According to the results of confusion matrixes of SVM models, the eight categories of each maize variety could be divided into three groups: Group 1 (0 h), Group 2 (12 and 24 h) and Group 3 (36, 48, 72, 96, 120 h). Maize kernels from different categories within one group were more likely to be misclassified with each other, and maize kernels within different groups had fewer misclassified samples. Germination test was conducted to verify the classification models, the results showed that the significant differences of maize kernel vigor revealed by standard germination tests generally matched with the classification accuracies of the SVM models. Hyperspectral imaging analysis for two varieties of maize kernels showed similar results, indicating the possibility of using hyperspectral imaging technique combined with chemometric methods to evaluate seed vigor and seed aging degree.

## 1. Introduction

Seeds enter an aging process after natural maturity. During this process, the vitality of the seed gradually decreases, which is a common phenomenon during the seed storage period. Seed vigor is an important indicator synthesizing seed germination, seedling rate, seedling growth potential, plant stress resistance and production potential [1,2]. For farmers, seeds with low viability will have low germination rates, which will increase their costs. Compared with seeds with low viability, seeds with high vigor which can save time, labor and material resources have obvious advantages [3]. Thus, an appropriate seed vigor detection method can help farmers engage in agricultural production activities in a better way. For seed companies, the seeds should be dried, processed and stored after harvest. If certain conditions are not suitable for seeds during these processes, it is possible to cause damage to the seeds, therefore, a rapid, non-destructive and high-accuracy method for seed vigor detection is of great help to them too.

The aging process of maize kernels can be influenced by maize varieties and environment factors such as temperature and humidity [4]. Generally, the natural aging of seeds is a long-duration procedure, which increases the cost of sampling for research purposes. In order to facilitate the research process, artificial accelerated aging tests are applied as a common method to simulate the seed aging procedure in a short time compared with natural aging. Studies have shown that artificial aging tests are an effective method to study seed vigor instead of natural aging. Han et al. identified quantitative trait loci (QTLs) for four maize seed vigor-related traits under artificial aging treatment [5]. Gelmond et al. applied accelerated aging to obtain six different levels of vigor of sorghum seeds from an identical lot [6]. Souza et al. also adopted an accelerated aging test during their study of the physiological quality of quinoa seeds under different storage conditions [7].

Most of the current research methods for seed aging determination are traditional physical and chemical detection methods. Mcdonough et al. studied the effects of accelerated aging on the vigor of maize, sorghum and sorghum powder. They detected both physical and chemical attributes that reveal the vitality of seeds. The density of maize and sorghum physical attribute was tested using a gas comparison pycnometer and tangential abrasive huller. The chemical attribute content of soluble protein in aged maize and sorghum was detected by gel chromatography with reagents [8]. Among all the seed vigor test methods, the standard germination test is the most widely used method for seed vigor detection, but it needs a complete sprouting procedure with the manual measurement of shoot length, root length and germination, which will take a long time. The disadvantages of traditional physical and chemical methods lies in that they are destructive, inefficient, time-consuming and usually involve complex operating procedures, thus a rapid, non-destructive method is needed for seed vigor detection.

Hyperspectral imaging technology is a new non-destructive test method which combines imaging information and spectral information [9,10,11,12,13]. Hyperspectral imaging can obtain the chemical information of heterogeneous samples and the spatial distribution of chemical components [14,15,16,17,18,19,20]. The hyperspectral imaging can be used to study the quality of seeds. Wei et al. used a visible/near-infrared hyperspectral imaging technique to detect the spatial distribution of aflatoxin B1 in kernels [21]. Wang et al. used hyperspectral imaging to predict the texture of maize seeds after different storage periods. The established quadrature signal correction-continuous new algorithm-piece partial least squares regression model (OSC-SPA-PLSR) had good prediction results of corn hardness and elasticity [22]. Williams et al. used near infrared (NIR) hyperspectral imaging to distinguish hard, intermediate and soft maize kernels from inbred lines. They used a Spectral Dimensions MatrixNIR camera and a short wave infrared (SWIR) hyperspectral imaging system to acquire the images of whole maize kernels. The authors used principal component analysis (PCA) to remove background, bad pixels, shading and found histological classes including glassy (hard) and floury (soft) endosperm on the cleaned images. They used PCA to discriminate endosperm from different kinds of maize kernels. Then PLS-DA was applied in classifying two kinds of maize. The result verified the effectiveness of the proposed method [10].

Hyperspectral imaging technology can also be used to detect the changes in seeds which underwent artificial accelerated aging test. Mcgoverin et al. investigated the viability of barley, wheat and sorghum grains using NIR hyperspectral imaging [11]. Nansen et al. adopted hyperspectral imaging to detect the germination rate of two native Australian tree species. During the process, hyperspectral images were acquired of individual seeds after 0, 1, 2, 5, 10, 20, 30 and 50 days of standard accelerated aging, and they found the loss of germination was associated with a significant change in seed coat spectral reflectance profiles [12]. Kandpal et al. predicted the viability of muskmelon seeds using NIR hyperspectral imaging system. After image collection, all seeds underwent a germination test to confirm their viability and vigor. The muskmelon seeds used in the study were vacuum-packed in plastic bags and stored in 45 °C hot water to age for 2, 4 and 6 days, while another set of seeds did not undergo artificial aging and were kept as the control (0 h). They found the spectral reflectance intensity decreases when there was an increment of seed viability, and this could reveal the changes in the chemical components in the seed as the artificial aging time increasing [13].

The main objective of this study was to explore the feasibility of using hyperspectral imaging to identify maize kernels vigor undergoing different accelerated aging time. The specific objectives were to: (1) conduct qualitative analysis of differences among maize kernels under different aging time by PCA; (2) build classification models and select optimal wavelengths to identify maize kernels undergoing different accelerated aging time; and (3) validate the results of hyperspectral imaging by standard germination tests.

## 2. Results and Discussion

### 2.1. Spectral Profile

**Figure 1:**
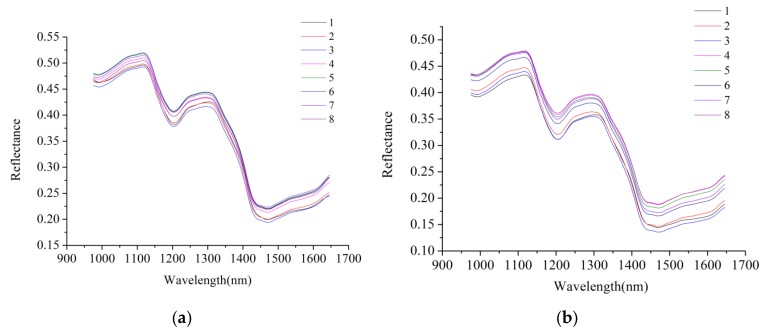
Average spectra of unprocessed spectra: (**a**) Maize 1; (**b**) Maize 2. Average spectra of maize kernels under different aging duration time differs in reflectance value.

The average spectral reflectance curves of maize kernels of two varieties at eight different aging times are shown in Figure 1. Similarity was observed in the change trends of the spectral reflectance curves of maize kernels which belonged to same variety but underwent different aging processes. The change trends of the reflectance curves of two varieties of maize showed clear similarities. Reflectance curves of maize kernels had differences in the reflectance of broad wavebands, so it was difficult to identify optimal wavelengths to discriminate maize kernels processed for different aging times.

**Figure 2:**
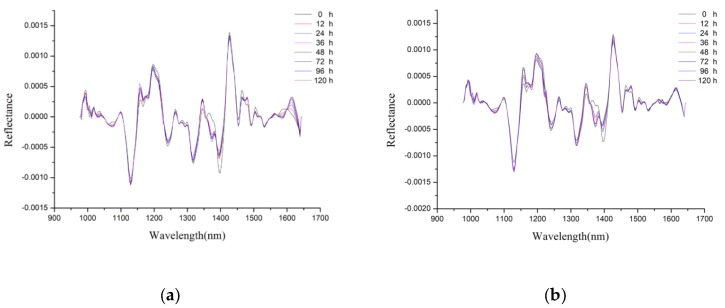
Average spectra preprocessed by second-order derivative: (**a**) Maize 1; (**b**) Maize 2. Spectral differences of maize kernels under different aging duration time at certain wavelengths could be observed.

The average spectra of two varieties of maize preprocessed by the second-order derivative with three smoothing points are shown in Figure 2. The second-order derivative spectra in Figure 2 show the main changes in the spectral reflectance among maize kernels for eight aging durations. The wavelengths with obvious difference in reflectance data were manually selected as the optimal wavelengths by comparing maize kernels exposed to the eight different aging treatments. The wavelengths showing obvious differences could be easily identified in Figure 2, and the interferences of unimportant wavelengths were greatly suppressed. The second-order derivative spectra showed more obvious differences of maize kernels among different aging time than unpreprocessed spectra. Moreover, it could be found in Figure 2 that the second-order derivative spectra of maize kernels under different aging time between two varieties of maize showed similar trends in their spectral curves.

### 2.2. PCA Analysis

#### 2.2.1. Pixel-Wise PCA Scores Visualization

One hyperspectral image under each aging time of each variety was randomly selected to conduct PCA analysis. The PCA score images of PC1, PC2 and PC3 of Maize 1 and Maize 2 were shown in Figures S1 and S2 of the Supplementary Materials, respectively. Using the original images as references, it can be seen in Figures S1 and S2 that warm colours (yellow-red) were related to soft endosperm, while cold colours (green-blue) were associated with hard endosperm. The compositional structure of unprocessed maize kernels was shown more clearly in score images, while the structure outline inside maize kernels after accelerated aging treatment were fuzzier. That might be because the accelerated aging treatment altered the physical and chemical attributes of material inside the seeds, causing the hardness to change to varying degrees in different parts of maize kernels.

#### 2.2.2. Object-Wise PCA Scores Scatter Plots Analysis

**Figure 3:**
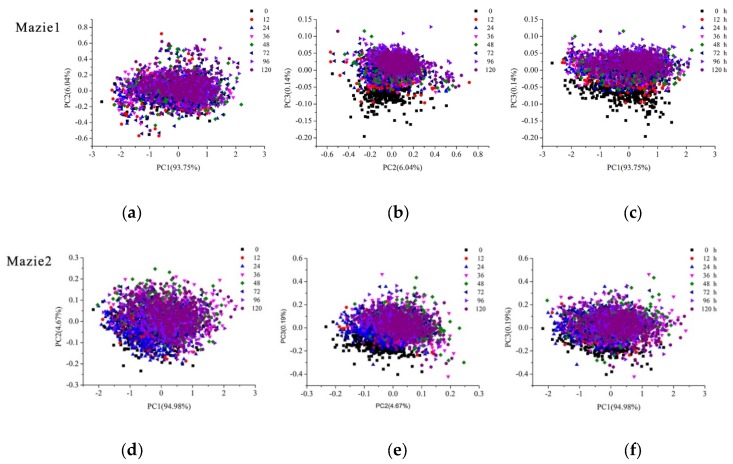
PCA scores scatter plots of maize kernels under different aging duration time: (**a**) PC1 versus PC2 for Maize 1; (**b**) PC2 versus PC3 for Maize 1; (**c**) PC1 versus PC3 for Maize 1; (**d**) PC1 versus PC2 for Maize 2; (**e**) PC2 versus PC3 for Maize 2; (**f**) PC1 versus PC3 for Maize 2. Clusters show the differences of maize kernels under different aging duration time.

PCA analysis was conducted on average spectra of maize kernels to explore the scores scatter of different PCs. The first three PCs for each kind of maize were used to conduct qualitative analysis because the first three PCs contained the most of information of maize kernel, with 99.98% explained variance for Maize 1 (93.75% for PC1, 6.04% for PC2 and 0.14% for PC3) and 99.84% for Maize 2 (94.98% for PC1, 4.67% for PC2 and 0.14% for PC3). According to Figure 3, maize kernels after different aging processing treatments were grouped together depending on different features of their own spectral characteristics though there were some overlapping among the eight clusters. Maize kernels without aging processing were partly separated on Figure 3b–e, which revealed that the differences among maize kernels without aging treatment and maize kernels under seven different aging treatments had more differences in hyperspectral imaging. In order to obtain satisfactory classification results, further processing should be conducted.

### 2.3. Classification Models Based on Full Spectra

PCA analysis indicated that differences existed among maize kernels after different aging times. SVM models were built to evaluate the differences among maize kernels exposed to different aging times. To build SVM models, the maize kernels of each category were randomly split into a calibration set and a prediction set at the ratio of 2:1 (400 maize kernels in the calibration set and 200 maize kernels in the prediction set).

The overall classification results of Maize 1 and Maize 2 are shown in Table 1. A SVM model using the spectra of the combination of Maize 1 and Maize 2 was also built, and the overall classification results also presented in Table 1. As shown in Table 1, the overall classification accuracy of the calibration sets for Maize 1 and Maize 2 was approximately 80%, but the prediction accuracy of Maize 1 was a little higher than that of Maize 2, with Maize 1 reaching 70% and Maize 2 only 60%. The SVM model using the combined dataset showed close classification results to the SVM models using Maize 1 and Maize 2.

To explore the details of the classification results of maize kernels under different aging times, Table 2 shows the confusion matrix of maize kernels of each category obtained according to the results of the SVM models using the full spectra. From Table 2, it could be seen that for Maize 1, Maize 2 and the combination of Maize 1 and Maize 2, maize kernels could be divided into three groups. The first group (Group 1) contained the maize kernels aged for 0 h, with nearly no misclassification with other categories. Because maize kernels aged for 12 and 24 h were more likely to be misclassified with each other, these two categories were sorted as Group 2, but the categories from Group 2, still had a high classification accuracy in both the calibration set and prediction set. The third group (Group 3) contained the maize kernels aged for 36, 48, 72, 96, 120 h. The maize kernels aged for each duration time in Group 3 gave lower classification accuracies, and they were grouped together because they were misclassified with each other more often.

From Table 2, although maize kernels within one group would be misclassified with each other, maize kernels were not easily misclassified with categories from the other groups. Table 3 showed the classification accuracy of SVM models of three groups (Group 1 (0 h), Group 2 (12 and 24 h) and Group 3 (36, 48, 72, 96, 120 h)) using full spectra. All accuracies were above 90%.

As shown in Table 1 and Table 2 the SVM model using the combined dataset showed close classification results to the SVM models using Maize 1 and Maize 2, and the general trend of classification accuracy of each aging duration time of the combined dataset was similar to Maize 1 and Maize 2. The results indicated that it would be possible to build a non-variety specific classification model for maize kernel vigor detection.

### 2.4. Optimal Wavelengths Selection

In this study, the second-order derivative was adopted to select the optimal wavelengths. As shown in Figure 2, the wavelengths with larger differences among maize kernels aged for different duration were highlighted in the spectra. The peaks and valleys with larger differences were selected as the optimal wavelengths to identify maize kernels at different aging times. The selected optimal wavelengths for Maize 1 and Maize 2 are shown in Table 4, and 19 and 18 optimal wavelengths were obtained finally to reduce the data volume. The optimal wavelengths near 995 nm were related to the second vibration of N–H bonds in proteins or amino acids [23]. The attributes of the secondary stretching vibration of C–H bonds in starch, proteins or lipids were revealed at optimal wavelengths near 1200 nm [24]. The spectral bands near 1463 nm were concerned with the absorption region of water [25]. From Table 4, the differences of the optimal wavelengths selected for Maize1 and Maize2 might be related to the genotypic differences for two varieties of maize.

### 2.5. Classification Models on the Optimal Wavelengths

The overall results of SVM models of Maize 1 and Maize 2 using the optimal wavelengths selected by second-order derivative spectra are shown in Table 5. For Maize 1, the classification accuracy of the calibration set was over 70%, and the classification accuracy of the prediction set was about 60%. For Maize 2, the classification accuracy of the calibration set reached 71%, and the classification accuracy of the prediction set was 62%. A slight decrease could be found between the overall results of the calibration set of SVM models using full spectra and optimal wavelengths, and the results of the prediction set were quite close to each other. The number of wavelengths reduced to 18 and 19 by optimal wavelengths selection, resulting in reduction of spectral data volume to 91% and 90.5%. During this process, some useful information was lost, leading to the accuracy reduction in SVM models based on optimal wavelengths. In the case of small difference between the classification results based on full spectra and optimal wavelengths, it was still meaningful to adopt the classification model based on optimal wavelengths.

To explore the classification results of maize kernels at different aging time using optimal wavelengths, the confusion matrix of maize kernels of each category obtained by SVM models using optimal wavelengths of Maize 1 and Maize 2 were shown in Table 6. Classification results of Maize 1 and Maize 2 using optimal wavelengths could also be divided into the three same groups as the SVM models using full spectra. Group 1 contained maize kernels under aging time of 0 h, Group 2 contained maize kernels under aging time of 12 and 24 h, and Group 3 contained maize kernels under the aging time of 36, 48, 72, 96 and 120 h. Maize kernels aged for different duration time within one group were more likely to be misclassified with each other. And maize kernels within different groups had fewer misclassified samples. Table 7 showed the classification accuracy of SVM models of three groups (Group 1 (0 h), Group 2 (12 and 24 h) and Group 3 (36, 48, 72, 96, 120 h)) using optimal wavelengths selected by second-order derivative spectra. Although the accuracies were slightly lower than models based on full spectra, the accuracies were still above 90%.

### 2.6. Germination Tests Analysis

A germination test was carried out to validate the accuracy of hyperspectral imaging in this study. Table 8 showed the germination rate, shoot length and root length of Maize 1 and Maize 2 at different aging duration. It could be seen that Maize 1 and Maize 2 at aging duration time from 0 to 24 h had significant differences in shoot length and root length, but there were no significant difference in germination rate among these three categories. Because the germination rate was calculated by seeds with at least 1 cm germ after 10 days, accelerated aging treatment for a short time (12 h and 24 h) may not affect the germination ability for maize kernels obviously, but the significant differences of root length and shoot length could reveal the vigor differences of maize kernels from these three categories, which consisted with the high classification accuracies of SVM models of Group 1 (0 h) and Group 2 (12 and 24 h). It also could be found in Table 8 that there were small significant differences among maize kernels under aging duration of 36, 48, 72, 96 and 120 h, which consisted with the low accuracies of SVM models of Group 3. The overall results indicated that hyperspectral imaging could be used to detect seed vigor under different aging duration time.

## 3. Materials and Methods

### 3.1. Sample Preparation

Two varieties of maize kernels cultivated by a commercial seed company (Jiudingjiusheng Seed Industrial Co., Ltd., Beijing, China) with breed numbers of 106101 and 7879 (in this work the names Maize 1 and Maize 2 were used to refer to maize varieties 106101 and 7879, respectively) instead of their original chemical names, complying with the company rules. The two varieties of maize were sown and harvested in the same experimental field simultaneously in 2016. For each variety, 4800 maize kernels were prepared for artificial accelerated aging. Before accelerating aging treatment, maize kernels were disinfected with 1% hypochlorous acid (HClO) solution for 20 min and then the maize kernels were naturally dried after being rinsed with distilled water. The 4800 maize kernels of each variety were randomly divided into eight categories (600 kernels in each category). One category was selected as control group (0 h) placed at room temperature (20 °C, 60% relative humidity) and the other 7 categorizes were used to conduct aging process under different aging time (12, 24, 36, 48, 72, 96 and 120 h). Then the maize kernels were aged in LH-150S artificial aging box (Ansheng Instrument Ltd., Zhengzhou, Henan, China) with temperature of 45 °C and relative humidity of 99%. After accelerated aging treatment, maize kernels were disinfected, rinsed with distilled water, naturally air-dried, and stored in Kraft paper bags. After the acquisition of hyperspectral images, maize kernels of each category were divided into 30 samples (20 kernels in each sample) for standard germination analysis.

### 3.2. Hyperspectral Imaging System

The experiment was carried out using a hyperspectral imaging system with the spectral range of 874–1734 nm, the spectral resolution of 5 nm and the spatial resolution of 320 × 256 pixels. The system consisted of an ImSpector N17E imaging spectrograph (Spectral Imaging Ltd., Oulu, Finland), a Xeva 992 camera (Xenics Infrared Solutions, Leuven, Belgium) equipped with an OLES22 lens (Spectral Imaging Ltd., Oulu, Finland), two 150 W tungsten halogen lamps (2900 Lightsource, Illumination Technologies Inc., Elbridge, NY, USA) that were symmetrically placed and served as the light source and a conveyer belt (Isuzu Optics Corp., Taiwan, China). The imaging system was controlled by the software (Xenics N17E, Isuzu Optics Corp.), which can be used to calibrated and analyze the images as well.

### 3.3. Hyperspectral Image Acquisition and Calibration

The maize kernels were placed on a black plate with a very low reflectivity, so it is easy to isolate maize kernels from the background. During the experiment, the exposure time of the camera was 3500 μs. The distance between the lens and the plate was adjusted to 17.9 cm, and the moving speed of the conveyer belt was set to 13.8 mm/s. The above adjustments were aimed at obtaining a clear image without distortion.

Two reference standards were used to calibrate the raw images (*I_raw_*). The dark reference image (*I_dark_*) was acquired by covering the lens with lens cap whose reflectivity is about 0%. The white reference image (*I_white_*) was collected from a piece of pure white Teflon board whose reflectivity is about 100%. The calibrated image (*I_c_*) could be calculated as Equation (1):

(1) $$\;I_{c}=\frac{I_{raw}-I_{dark}}{I_{white}-I_{dark}}\;$$

### 3.4. Spectral Reflectance Extraction and Preprocessing

After image calibration, the spectral reflectance of each maize kernel was extracted from the hyperspectral images. Hyperspectral imaging provides spectral reflectance data and grayscale images at each wavelength. Prior to image processing, the maize kernels were separated from the background by using a mask 14, 26, 27. In this study, the mask was built by conducting image binaryzation on the gray-scale image at 1116 nm to set maize kernel area as 1 and the background as 0. The maize kernels were then isolated from background by applying the mask to the gray-scale images at each wavelength. Then, calibrated hyperspectral images were pre-processed to minimize noise 11, 28, 29. The original pixel-wise spectra were denoised by the wavelet transformation with decomposition level 3 using Daubechies 8 (db8) as the wavelet basis function. Then, the pixel-wise spectra of all pixels within a maize kernel were averaged as one spectrum.

### 3.5. Standard Germination Tests

Standard germination tests for Maize 1 and Maize 2 were conducted on ten kernels of each sample after acquiring hyperspectral images. For each sample, 10 maize kernels were picked randomly for germination tests. To obtain the vigor of maize kernels, the standard germination tests were performed according to the guidelines of the International Seed Testing Association (ISTA)30. Maize kernels were placed in round holes of sponges, and sponges were placed in seedling basins with enough water. Then all the seedling basins were stored in germination cabinet at 25 °C with 99% relative humidity for 10 days. According to ISTA standards, seeds with 1 cm germ after germination were considered to be seeds with viability. After germination, the germination percentage, shoot length and root length were calculated and measured manually.

### 3.6. Data analysis Methods

#### 3.6.1. Principal Component Analysis

Principal component analysis (PCA) is a multivariate statistical method that studies the correlation between multiple variables. It examines how a few principal components can be used to reveal the internal relationship between multiple variables. PCA derives principal components from the original variables, and the first few principal components (PCs) contained most of the useful information. The PCs are linear combinations of original variables, and they are orthotropic and irrelevant to each other. The scores of the first few PCs can be used to explore the differences between samples [24,25,26].

For hyperspectral images, there are two approaches to conduct PCA analysis, pixel-wise analysis and objective-wise analysis [15]. Pixel-wise analysis is to form PCA scores visualization image. For this method, PCA is calculated on individual pixels of the images. Scores of each pixel within the hyperspectral image of each PC can be obtained to form a scores visualization image. The differences between samples can be visually observed and explored in colormaps for each PC.

Object-wise analysis is to form PCA scores scatter plots. For this method, average spectra of the depicted objects are used instead of individual pixels. The scores of different PCs of samples are scattered in a two-dimensional space or a three-dimensional space. The differences among samples can be explored more clearly in these spaces [15,27].

#### 3.6.2. Optimal Wavelength Selection

The spectral data obtained by hyperspectral images usually have a large data volume and contain a lot of useless information like redundant and collinear information. The existence of useless information will increase the data processing burden, which is likely to casue instability of the model and thus result in a poor performance. Meanwhile, processing of a large amount of data places a high burden on computer hardware and increases the calculation time. Thus, it is necessary to select optimal wavelengths to reduce the inputs, which can simplify the model and improve the model performance. The second derivative is an efficient preprocessing method in spectral data analysis. It can eliminate the interference of other backgrounds, improve the spectral resolution and highlight useful information in the spectra. Differences in peaks and valleys of spectra preprocessed by second-order derivative indicate the physical and chemical changes of the samples, which has been used as an efficient method to identify optimal wavelengths [28,29]. Peaks and valleys with large differences in second-order derivative spectra can be selected as the optimal wavelengths.

#### 3.6.3. Discriminant Model

Support vector machine (SVM) is a supervised machine learning model used for classification and regression. The main idea of SVM is to create a hyperplane as a decision surface, which maximizes the margins of isolation between different samples. SVM could deal with both linear and nonlinear data efficiently with its good generalization ability. Kernel function is important for conducting SVM, and radial basis function (RBF) is a widely used kernel function. The parameters for SVM models should be determined, including the regularization parameter c and kernel function parameter g. The former determines the tradeoff between minimizing the training error and minimizing model complexity, and the latter defines the non-linear mapping from input space to some high dimensional feature space. The search range for c and g ranged from 2^−8^ to 2^8^ in this study. The optimal combination of c and g was determined by the SVM model with the highest classification accuracy. Grid-search was applied to optimize the two parameters for SVM in this study [30,31,32].

#### 3.6.4. Significance Test

Duncan’s multiple range tests were applied to calculate for comparison of maize vitality index (germination, shoot and root length) at different accelerated aging duration time at a significance level of 0.05 [33].

## 4. Conclusions

Hyperspectral imaging technology combined with SVM models was used to identify the vigor of maize kernels after different aging times. The results of SVM models based on optimal wavelengths were about 10% lower than that of models based on full spectra. However, it was meaningful to conduct optimal wavelengths selection because of the obvious improvement in modeling speed. Confusion matrixes for maize kernels of each category were built for both SVM models using full spectra and optimal wavelengths to reveal the detail of classification results of maize kernels processed under different aging duration. From the results of confusion matrixes, 8 categories of maize kernels could be divided into three groups. Group 1 contained unprocessed maize kernels, Group 2 contained maize kernels aged for 12 and 24 h and Group 3 contained maize kernels with longer aging times (36, 48, 72, 96, 120 h). Maize kernels aged for different durations within one group were more likely to be misclassified with each other. Maize kernels within different groups had fewer misclassified samples. To verify the results of SVM models, traditional seed vigor testing method, standard germination test was conducted. The results of standard germination tests were generally consistent with those of SVM models. Maize kernels belonging to Group 1 (0 h) and Group 2 (12 h and 24 h) had significant differences for root length and shoot length. Maize kernels belonging to Group 3 (36 h, 48 h, 72 h, 96 h and 120 h) had no significant differences with each other comprehensively considering germination rate, root length and shoot length.

The results of this research demonstrate that it is feasible to detect maize kernel vigor with a hyperspectral imaging system combined with SVM models and the second-order derivative spectra could be used to select optimal wavelengths which do great help in shortening modeling time. Thus, as a rapid, non-destructive method, hyperspectral imaging system has great potential for application in seed vigor detection. To improve model performances, different varieties of maize kernels from different crop years, growth locations and storage conditions will be take into consideration to extend the database in the future researches. Variety specific models and non-variety specific models will also be explored for real-world application.

## Figures and Tables

**Table 1:** The classification accuracy of SVM models using full spectra.

Sample Variety	C ^1^	G ^2^	Cal. ^3^ (%)	Pre. ^4^ (%)	Cv. ^5^
Maize 1	256.00	1.74	81.53	68.15	58.13
Maize 2	256.00	3.03	78.47	60.16	63.84
Maize Mixed	256.00	5.28	73.43	59.90	57.23

^1^ The regularization parameter of SVM; ^2^ The kernel function parameter of SVM; ^3^ Calibration set; ^4^ Prediction set; ^5^ Five-fold cross-validation.

**Table 2:** Confusion matrix of SVM models using full spectra.

Sample Variety		Sample Number	Pre.								Accuracy (%)
			1 ^1^	2	3	4	5	6	7	8	
Maize 1	Cal.	1 (400)	400	0	0	0	0	0	0	0	100.00
		2 (400)	0	384	16	0	0	0	0	0	96.00
		3 (400)	0	14	356	26	3	0	1	0	89.00
		4 (400)	0	3	34	306	40	11	5	1	76.50
		5 (400)	0	0	16	74	228	68	2	12	57.00
		6 (400)	0	0	4	25	76	261	8	26	65.30
		7 (400)	0	0	0	1	5	18	327	49	81.80
		8 (400)	0	0	0	5	9	20	19	347	86.80
	Pre.	1 (200)	199	1	0	0	0	0	0	0	99.50
		2 (200)	1	150	47	0	0	2	0	0	75.00
		3 (200)	0	19	158	17	6	0	0	0	79.00
		4 (200)	0	4	26	114	39	16	0	1	57.00
		5 (200)	0	0	2	11	92	87	0	8	46.00
		6 (199)	0	1	2	22	66	100	3	5	50.30
		7 (200)	0	0	0	1	10	12	117	60	58.50
		8 (199)	0	0	0	1	6	17	16	159	79.90
Maize 2	Cal.	1 (400)	400	0	0	0	0	0	0	0	100.00
		2 (400)	0	374	24	0	0	0	2	0	93.50
		3 (400)	0	16	384	0	0	0	0	0	96.00
		4 (400)	0	0	0	279	37	27	10	47	69.80
		5 (400)	0	0	0	38	322	6	0	34	80.50
		6 (400)	0	1	0	30	2	256	95	16	64.00
		7 (400)	0	1	0	17	1	105	259	17	64.80
		8 (400)	0	1	0	79	53	22	8	237	59.30
	Pre.	1 (200)	196	0	3	0	0	0	0	1	98.00
		2 (200)	1	156	36	0	0	1	6	0	78.00
		3 (199)	1	21	177	0	0	0	0	0	88.90
		4 (200)	0	2	0	90	36	23	10	39	45.00
		5 (200)	1	0	0	47	109	4	2	37	54.50
		6 (200)	0	3	0	12	3	80	92	10	40.00
		7 (200)	0	6	1	19	0	71	93	10	46.50
		8 (200)	0	1	0	71	41	16	10	61	30.50
Maize mixed	Cal.	1 (800)	800	0	0	0	0	0	0	0	100.00
		2 (800)	0	654	134	0	0	8	0	4	81.80
		3 (800)	0	123	657	17	1	2	0	0	82.10
		4 (800)	0	5	30	499	126	35	40	65	62.40
		5 (800)	0	4	19	156	422	129	22	48	52.80
		6 (800)	0	7	4	52	70	480	110	77	60.00
		7 (800)	0	0	0	57	51	125	409	158	51.00
		8 (800)	0	2	1	40	64	75	87	531	66.40
	Pre.	1 (400)	394	2	3	0	0	1	0	0	98.50
		2 (400)	4	246	130	0	2	11	3	4	61.50
		3 (399)	2	94	287	9	1	4	0	2	72.20
		4 (400)	0	9	16	205	90	26	25	29	51.30
		5 (400)	0	3	3	55	136	130	20	53	34.00
		6 (399)	0	3	5	36	64	187	74	30	46.90
		7 (400)	0	3	4	36	59	167	77	54	19.30
		8 (399)	0	3	0	40	59	51	58	188	47.10

^1^ 1, 2, 3, 4, 5, 6, 7 and 8 are assigned respectively as the category value of the maize kernels processed under different aging duration (12, 24, 36, 48, 72, 96 and 120 h).

**Table 3:** The classification accuracy of SVM models of three groups using full spectra.

Sample Variety		Sample Number	Pre.			Accuracy (%)
			Group 1	Group 2	Group 3	
Maize 1	Cal.	Group 1 (400)	400	0	0	100.00
		Group 2 (800)	0	770	30	96.25
		Group 3 (2000)	0	57	1943	97.15
	Pre.	Group 1 (200)	199	1	0	99.50
		Group 2 (400)	1	374	25	93.50
		Group 3 (998)	0	35	963	96.49
Maize 2	Cal.	Group 1 (400)	400	0	0	100.00
		Group 2 (800)	0	798	2	99.75
		Group 3 (2000)	0	3	1997	99.85
	Pre.	Group 1 (200)	196	3	1	98.00
		Group 2 (399)	2	390	7	97.74
		Group 3 (1000)	1	13	986	98.60
Maize mixed	Cal.	Group 1 (800)	800	0	0	100.00
		Group 2 (1600)	0	1568	32	98.00
		Group 3 (4000)	0	72	3928	98.20
	Pre.	Group 1 (400)	394	5	1	98.50
		Group 2 (799)	6	757	36	94.74
		Group 3 (1998)	0	49	1949	97.55

**Table 4:** Corresponding optimal wavelengths selected by second-order derivative spectra.

Sample Variety	No.	Optimal Wavelengths (nm)
Maize 1	19	995, 1005, 1035, 1076, 1130, 1156, 1167, 1207, 1241, 1264, 1321, 1375, 1399, 1426, 1463, 1480, 1504, 1585, 1615
Maize 2	18	1005, 1072, 1130, 1156, 1160, 1167, 1197, 1241, 1264, 1318, 1345, 1372, 1396, 1426, 1453, 1463, 1480, 1612

**Table 5:** The classification accuracy of SVM models using the optimal wavelengths selected by second-order derivative spectra.

Sample Variety	c	g	Cal. (%)	Pre. (%)	Cv.
Maize 1	256.00	27.86	70.47	57.45	71.31
Maize 2	256.00	16.00	71.66	62.48	63.81

**Table 6:** Confusion matrix of SVM models using optimal wavelengths selected by second-order derivative spectra.

Sample Variety		Sample Number	Prediction Value								Accuracy (%)
			1	2	3	4	5	6	7	8	
Maize 1	Cal.	1 (400)	400	0	0	0	0	0	0	0	100.00
		2 (400)	0	372	27	1	0	0	0	0	93.00
		3 (400)	0	33	314	14	23	12	1	3	78.50
		4 (400)	0	5	27	190	57	68	11	42	47.50
		5 (400)	0	3	43	54	196	66	3	35	49.00
		6 (400)	0	0	17	92	82	170	3	36	42.50
		7 (400)	0	0	0	19	7	13	319	42	79.80
		8 (400)	0	1	5	31	29	26	14	294	73.50
	Pre.	1 (200)	199	1	0	0	0	0	0	0	99.50
		2 (200)	2	161	36	0	0	1	0	0	80.50
		3 (200)	0	36	131	6	14	6	0	7	65.50
		4 (200)	0	4	31	66	41	47	1	10	33.00
		5 (200)	0	2	25	37	77	37	2	20	38.50
		6 (199)	0	2	13	62	53	43	4	22	21.60
		7 (200)	0	0	0	11	10	10	121	48	60.50
		8 (199)	0	0	0	21	22	16	20	120	60.30
Maize 2	Cal.	1 (400)	400	0	0	0	0	0	0	0	100.00
		2 (400)	0	365	33	0	0	2	0	0	91.30
		3 (400)	0	25	375	0	0	0	0	0	93.80
		4 (400)	0	0	0	246	54	29	10	61	61.50
		5 (400)	0	0	0	57	295	6	0	42	73.80
		6 (400)	0	5	0	38	1	230	113	13	57.50
		7 (400)	0	2	0	21	1	165	196	15	49.00
		8 (400)	0	0	0	117	63	27	7	186	46.50
	Pre.	1 (200)	196	0	3	0	0	0	0	1	98.00
		2 (200)	1	164	30	0	0	2	3	0	82.00
		3 (199)	2	15	182	0	0	0	0	0	91.50
		4 (200)	0	0	0	86	35	11	11	57	43.00
		5 (200)	1	0	0	36	126	3	2	32	63.00
		6 (200)	0	2	0	16	2	93	84	3	46.50
		7 (200)	0	2	0	20	0	75	94	9	47.00
		8 (200)	0	0	0	78	46	12	6	58	29.00

**Table 7:** The classification accuracy of SVM models of three groups using optimal wavelengths selected by second-order derivative spectra.

Sample Variety		Sample Number	Pre.			Accuracy (%)
			Group 1	Group 2	Group 3	
Maize 1	Cal.	Group 1 (400)	400	0	0	100.00
		Group 2 (800)	0	746	54	93.25
		Group 3 (2000)	0	101	1899	94.95
	Pre.	Group 1 (200)	199	1	0	99.50
		Group 2 (400)	2	364	34	91.00
		Group 3 (998)	0	77	921	92.28
Maize 2	Cal.	Group 1 (400)	400	0	0	100.00
		Group 2 (800)	0	798	2	99.75
		Group 3 (2000)	0	7	1993	99.65
	Pre.	Group 1 (200)	196	3	1	98.00
		Group 2 (399)	3	391	5	97.99
		Group 3 (1000)	1	4	995	99.50

**Table 8:** Germination rate, shoot and root length of Maize 1 and Maize 2 under different accelerated aging time.

Sample Variety	Accelerating Aging Time (hrs)	Germination Rate (%)	Shoot Length (cm/seedling)	Root Length (cm/seedling)
Maize 1	0	92.00a	11.30a	23.15a
	12	90.67a	12.26b	21.42b
	24	86.00a	9.77c	17.31cd
	36	75.33b	6.35d	18.20c
	48	73.67b	8.95c	16.68d
	72	76.33b	6.17d	13.76e
	96	59.00c	5.60d	12.80e
	120	57.00c	5.99d	12.69e
Maize 2	0	96.33a	13.06a	24.68a
	12	97.67a	10.64b	24.11a
	24	93.00a	10.09bc	19.25b
	36	82.67b	8.78cd	17.08c
	48	79.00bc	6.98e	18.46b
	72	75.33c	7.27de	12.80d
	96	62.00d	5.93e	14.63e
	120	63.67d	6.73e	12.13e

The letters (a, b, c, d, e) in each column indicate the significance of difference among maize kernel processed by different duration of aging time at the confidence level of 5% (Duncan’s). Within a column, data followed by different letters are significantly different.

## Supplementary Materials

The following are available online, Figure S1: Score images for the first three principal components of Maize 1: (**a**) Score image for PC1. (**b**) Score image for PC2. (**c**) Score image for PC3. The color bar indicates the score value of each pixel, differences of maize kernels under different accelerating aging duration time could be seen according to the score images. Warm color (positive score values) were related to soft endosperm, while cold color (negative score values) were associated with hard endosperm; Figure S2: Score images for the first three principal components of Maize 2: (**a**) Score image for PC1. (**b**) Score image for PC2. (**c**) Score image for PC3. The color bar indicates the score value of each pixel, differences of maize kernels under different accelerating aging duration time could be seen according to the score images. Warm color (positive score values) were related to soft endosperm, while cold color (negative score values) were associated with hard endosperm.
