# Supplementary material for: Identification of Maize Kernel Vigor under Different Accelerated Aging Times Using Hyperspectral Imaging
Source: Molecules. 2018 Nov 25;23(12):3078. doi: 10.3390/molecules23123078 (PMC6321087; doi:10.3390/molecules23123078)
Supplement: Supplementary file 1 [file molecules-23-03078-s001.pdf]

Supplementary file

Figure captions

Figure S1 Score images for the first three principal components of Maize 1: (a) Score image for PC1. (b) Score image for PC2. (c) Score image for PC3. The color bar indicates the score value of each pixel, differences of maize kernels under different accelerating aging duration time could be seen according to the score images. Warm color (positive score values) were related to soft endosperm, while cold color (negative score values) were associated with hard endosperm.

Figure S2 Score images for the first three principal components of Maize 2: (a) Score image for PC1. (b) Score image for PC2. (c) Score image for PC3. The color bar indicates the score value of each pixel, differences of maize kernels under different accelerating aging duration time could be seen according to the score images. Warm color (positive score values) were related to soft endosperm, while cold color (negative score values) were associated with hard endosperm.

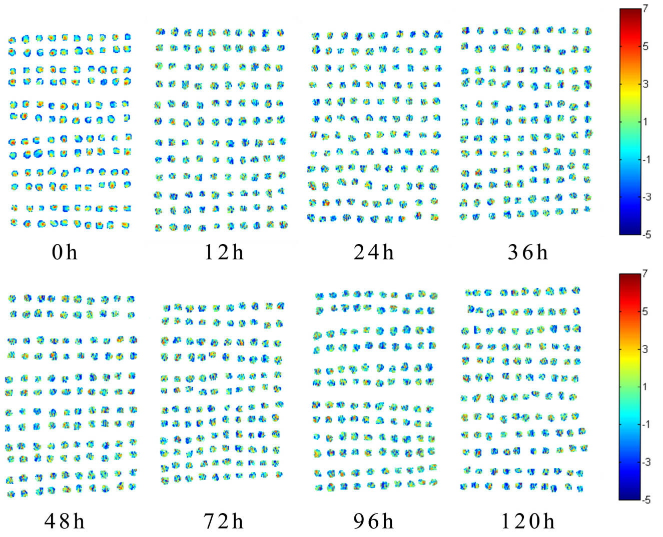
0h
12h
24h
36h
48h
72h
96h
120h
7
5
3
1
-1
-3
-5

(a)

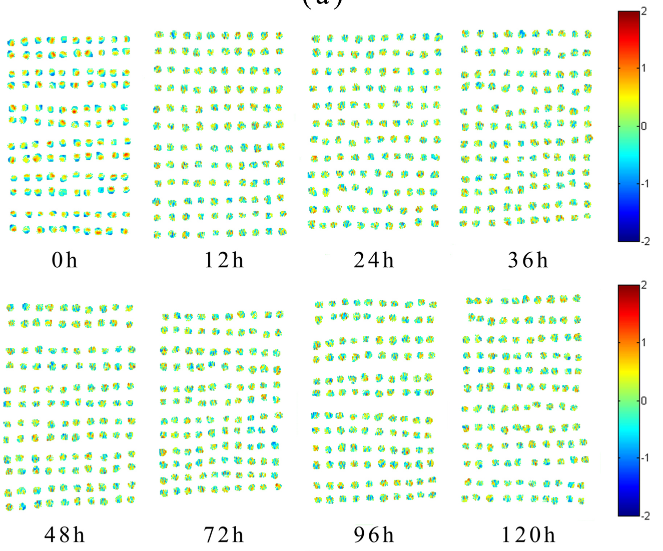
0h
12h
24h
36h
48h
72h
96h
120h
2
1
0
-1
-2

(b)

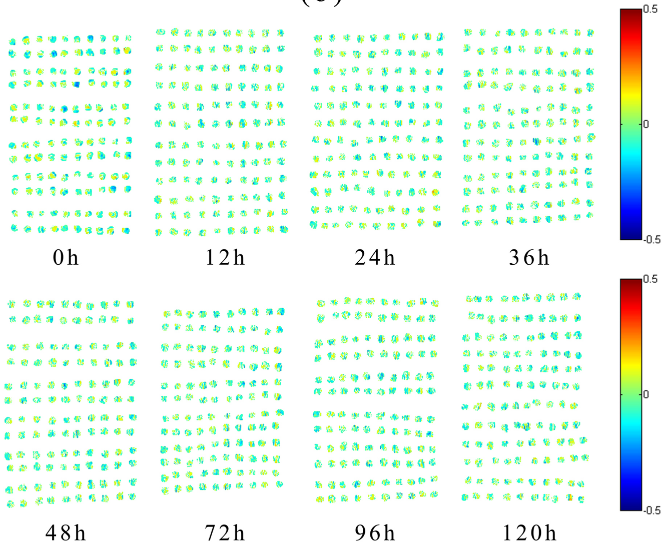
0h
12h
24h
36h
48h
72h
96h
120h
0.5
0
-0.5

(c)

Figure S1 Score images for the first three principal components of Maize 1: (a) Score image for PC1. (b) Score image for PC2. (c) Score image for PC3. The color bar indicates the score value of each pixel, differences of maize kernels under different accelerating aging duration time could be seen according to the score images. Warm color (positive score values) were related to soft endosperm, while cold color (negative score values) were associated with hard endosperm.

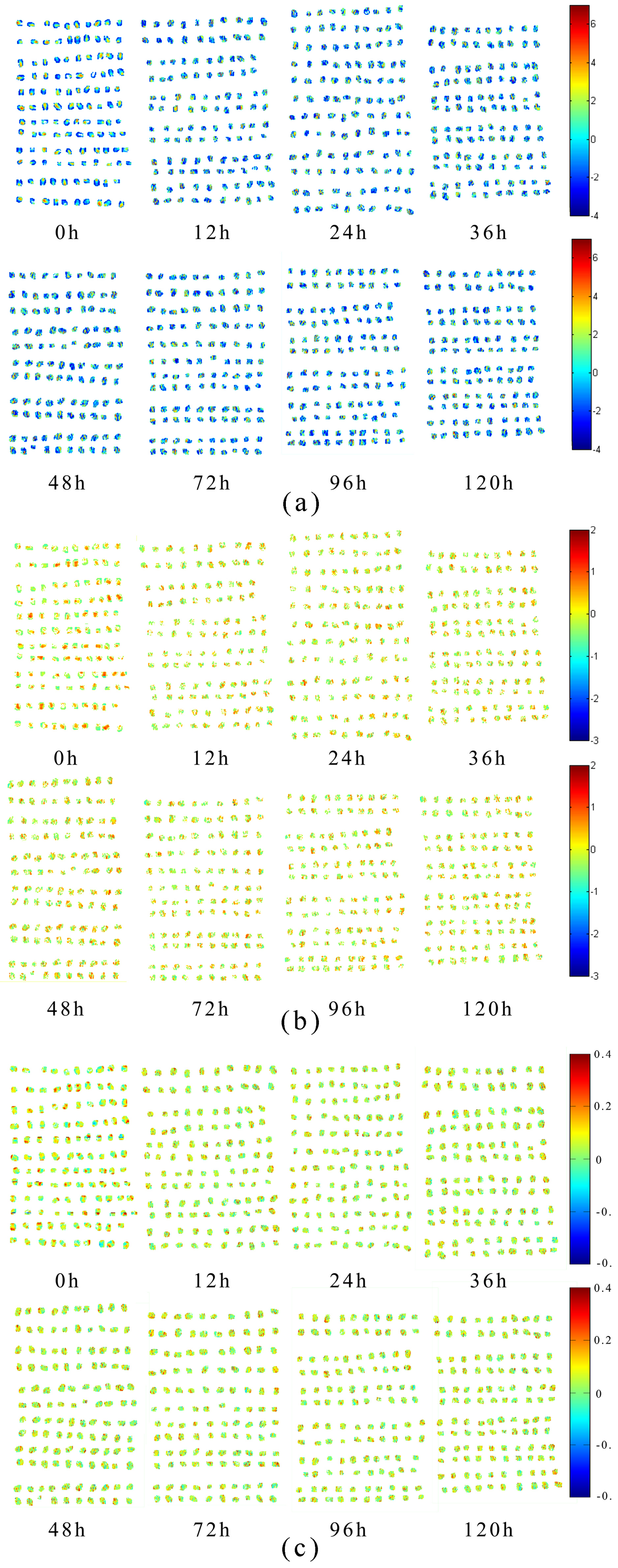
6
4
2
0
-2
-4
0h
12h
24h
36h
48h
72h
96h
120h
(a)
2
1
0
-1
-2
-3
0h
12h
24h
36h
48h
72h
96h
120h
(b)
0.4
0.2
0
-0.
-0.
0h
12h
24h
36h
48h
72h
96h
120h
(c)

Figure S2 Score images for the first three principal components of Maize 2: (a) Score image for PC1. (b) Score image for PC2. (c) Score image for PC3. The color bar indicates the score value of each pixel, differences of maize kernels under different accelerating aging duration time could be seen according to the score images. Warm color (positive score values) were related to soft endosperm, while cold color (negative score values) were associated with hard endosperm.
